# Supplementary material for: Artificial intelligence reveals past climate extremes by reconstructing historical records
Source: Nat Commun. 2024 Oct 24;15:9191. doi: 10.1038/s41467-024-53464-2 (PMC11502677; doi:10.1038/s41467-024-53464-2)
Supplement: Supplementary file 1 — Supplementary Information [file 41467_2024_53464_MOESM1_ESM.pdf]

Supplementary information to Artificial Intelligence Reveals Past Climate Extremes by Reconstructing Historical Records

Étienne Plésiat *[1], Robert J. H. Dunn[2], Markus G. Donat[3,4], and Christopher Kadow[1]

[1]German Climate Computing Center (DKRZ), Bundestraße, 45a, Hamburg, 20146, Germany
[2]Met Office Hadley Centre, Fitzroy Rd, Exeter, EX1 3PB, United Kingdom
[3]Barcelona Supercomputing Center (BSC), Plaça d'Eusebi Güell, 1-3, Barcelona, 08034, Spain
[4]Institució Catalana de Recerca i Estudis Avançats (ICREA), Barcelona, Spain

Table S1: **Overview of the number of samples, temporal coverage and utilization of the datasets.** The temporal coverage reported here corresponds to the time period used in the study. Ballot symbols indicates the usage of each dataset for three specific tasks: training of the AI model, evaluation of the AI model, analysis of the dataset. Notably, the CMIP6 data used for training, validation and evaluation are distinct.

	Samples	Time span	Training	Validation	Evaluation	Analysis
**CMIP6**	61560	1901-2014	✗	✗	✗	
**ERA5**	948	1940-2018			✗	✗
**HadEX-CAM**	1416	1901-2018			✗	✗
**HadEX3**	1416	1901-2018				✗
**20crV3**	1380	1901-2015				✗

*plesiat@dkrz.de

Table S2: **List of CMIP6 models and members used to create the training, validation and test datasets.**

Model	Member
AWI-CM-1-1-MR	r1i1p1f1, r2i1p1f1, r3i1p1f1, r4i1p1f1, r5i1p1f1, r1i1p1f2
CNRM-CM6-1-HR	r1i1p1f2
EC-Earth3-AerChem	r1i1p1f1
EC-Earth3-CC	r1i1p1f1
EC-Earth3	r1i1p1f1, r2i1p1f1, r3i1p1f1, r4i1p1f1, r6i1p1f1, r7i1p1f1, r9i1p1f1, r10i1p1f1, r11i1p1f1, r12i1p1f1, r14i1p1f1, r15i1p1f1, r16i1p1f1, r17i1p1f1, r18i1p1f1, r19i1p1f1, r20i1p1f1, r21i1p1f1, r22i1p1f1, r23i1p1f1, r24i1p1f1, r25i1p1f1
EC-Earth3-Veg	r1i1p1f1
HadGEM3-GC31-MM	r1i1p1f3, r2i1p1f3, r3i1p1f3, r4i1p1f3
MPI-ESM1-2-HR	r1i1p1f1, r2i1p1f1, r3i1p1f1, r4i1p1f1, r5i1p1f1, r6i1p1f1, r7i1p1f1, r8i1p1f1, r9i1p1f1, r10i1p1f1

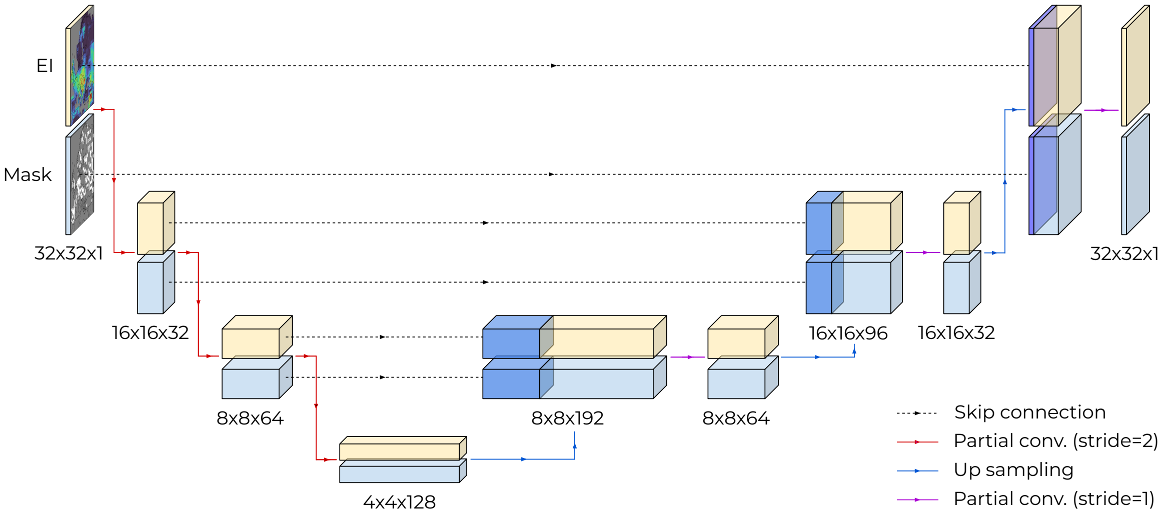

Figure S1: **Architecture of the neural network used to reconstruct the extreme indices.** The network consists of an encoding and a decoding branch made of partial convolutional layers that process the input data (maps of extreme indices (EI) and masks of missing values) until all the missing values are reconstructed. Skip connections are utilized to retain spatial context and feature information across the network by concatenating the feature maps from the encoding layers to their corresponding decoding layers (as indicated by the dark blue boxes). Nearest neighbor upsampling is employed in each decoding layer to progressively increase the spatial resolution of the feature maps until the initial resolution is reached.

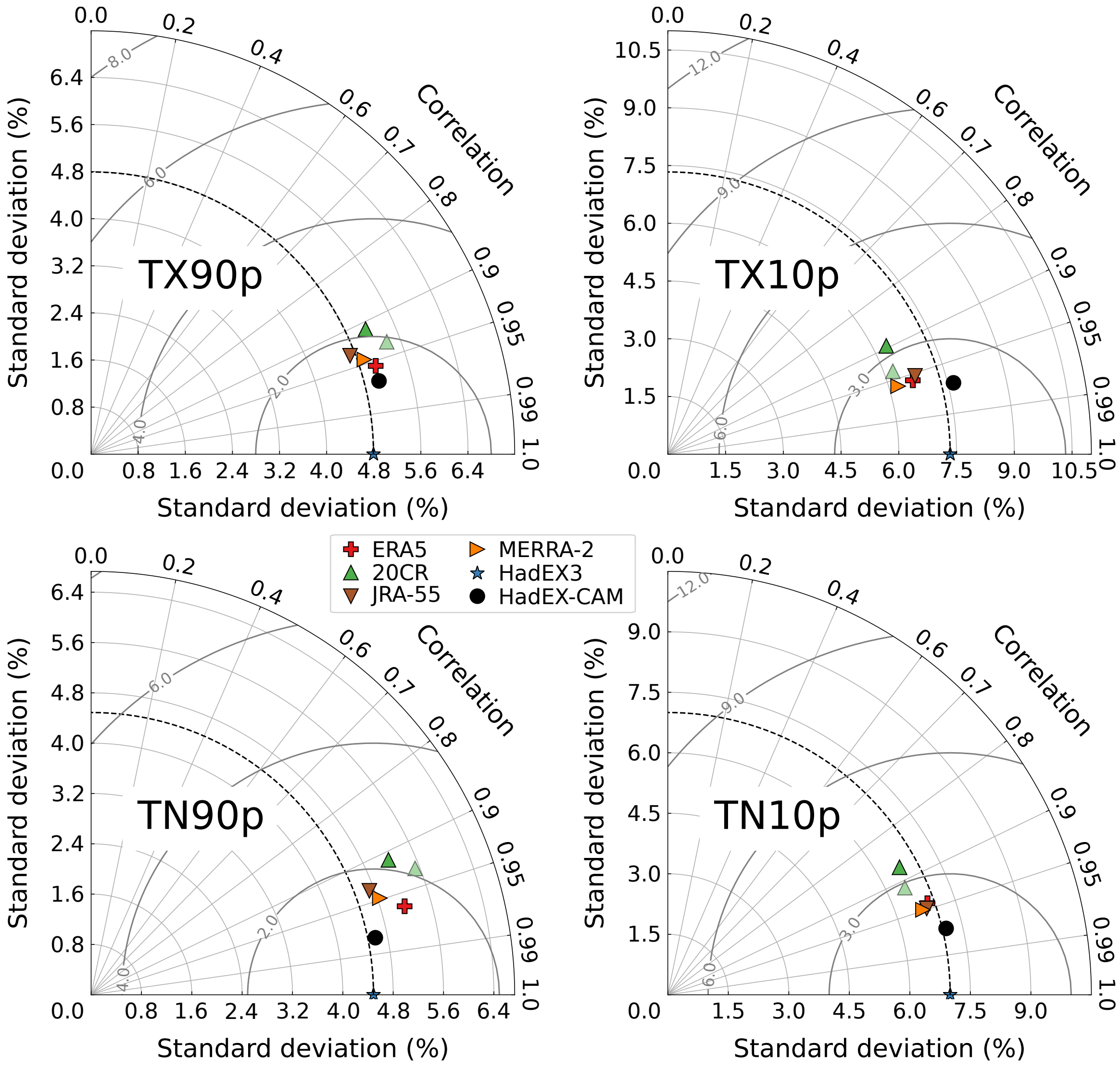

Figure S2: **Taylor diagrams for TX90p, TX10p, TN90p, TN10p using HadEX3 as a reference dataset.** TX90p is the percentage of days when the daily maximum temperature $> 90^{th}$ percentile. TX10p is the percentage of days when the daily maximum temperature $< 10^{th}$ percentile. TN90p is the percentage of days when the daily minimum temperature $> 90^{th}$ percentile. TN10p is the percentage of days when the daily minimum temperature $< 10^{th}$ percentile. The radial axis denotes the standard deviation (in %) of the time series, while the polar axis indicates the correlation. Semicircles centered on the reference dataset convey information regarding the root mean square error (RMSE). Comparisons with HadEX3 have been established for different time periods: 1901-2018 for HadEX-CAM (black circle), 1940-2018 for ERA5 (red cross), 1901-2018 for 20cr-v3 (green up-triangle), 1940-2018 for 20cr-v3 (semi-transparent green up-triangle), 1970-2018 for JRA-55 (brown down-triangle) and 1980-2018 for MERRA-2 (orange right-triangle).

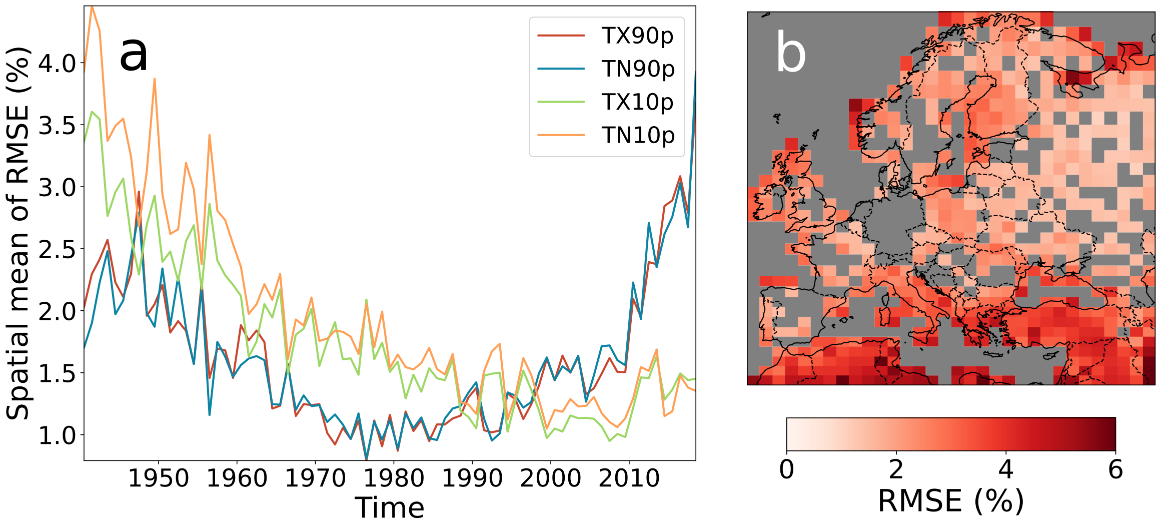

Figure S3: **Temporal and spatial root mean square error (RMSE) for the reconstruction of ERA5 using CRAI.** The two plots present the RMSE of the CRAI reconstruction of the masked version of ERA5 with respect to the original ERA5 dataset. a) shows the spatial mean of the RMSE for TX90p, TN10p, TX10p and TN10p, considering the reconstructed values only. TX90p is the percentage of days when the daily maximum temperature $> 90^{\text{th}}$ percentile. TX10p is the percentage of days when the daily maximum temperature $< 10^{\text{th}}$ percentile. TN90p is the percentage of days when the daily minimum temperature $> 90^{\text{th}}$ percentile. TN10p is the percentage of days when the daily minimum temperature $< 10^{\text{th}}$ percentile. b) shows the temporal mean of the RMSE averaged over the four extreme indices, considering the grid boxes with at least 10% of reconstructed values (otherwise, grid boxes are shown in gray).

Table S3: **Mean values and standard deviations (SD) calculated for the reference datasets used in the evaluations and for each extreme index.**

	**TX90p**		**TX10p**		**TN90p**		**TN10p**	
Dataset	Mean	SD	Mean	SD	Mean	SD	Mean	SD
**Test dataset**	8.57	11.07	14.63	15.52	8.23	10.32	15.16	16.00
**ERA5**	9.39	11.08	11.96	12.93	9.41	10.90	12.29	13.13
**HadEX-CAM**	9.34	10.90	12.13	12.75	8.72	9.72	12.41	12.36

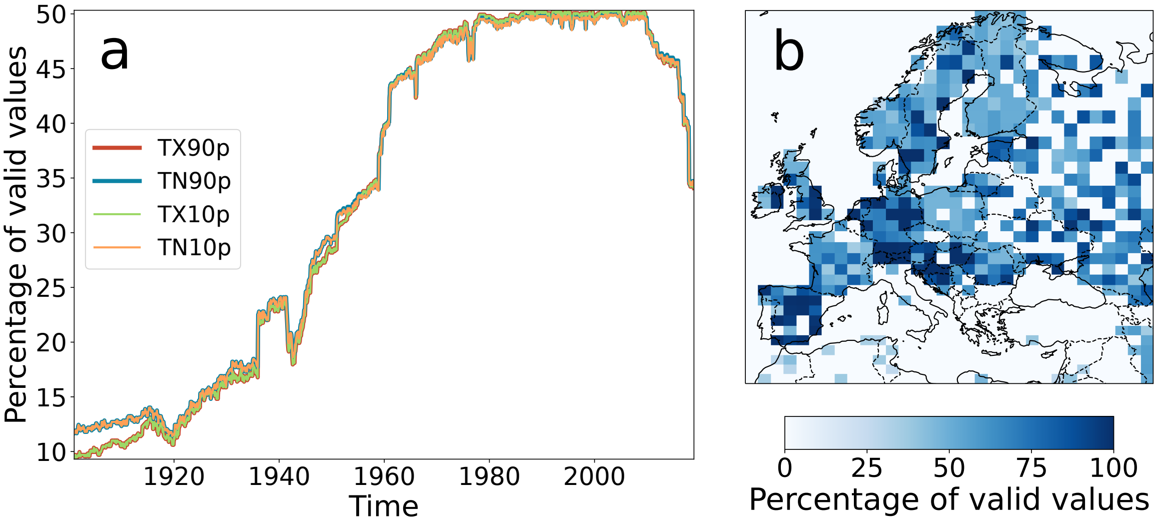

Figure S4: **Percentage of valid values in the original HadEX-CAM dataset.** a) Time evolution of the percentage of valid values with respect to the complete land dataset for TX90p, TN10p, TX10p and TN10p. TX90p is the percentage of days when the daily maximum temperature $> 90^{th}$ percentile. TX10p is the percentage of days when the daily maximum temperature $< 10^{th}$ percentile. TN90p is the percentage of days when the daily minimum temperature $> 90^{th}$ percentile. TN10p is the percentage of days when the daily minimum temperature $< 10^{th}$ percentile. b) Spatial distribution of the percentage of valid values averaged over all extreme indices. 100% of valid values corresponds to the total number of values per month in the reconstructed HadEX-CAM dataset, i.e. 763 values.

Table S4: **Evaluation of the reconstruction methods using the test dataset.** The table shows the Wasserstein distance WD (left inner column) and the coefficient of determination R^2 (right inner column) calculated on the reconstructed values only for each extreme index and for four datasets: the reconstruction of a masked version of the HadEX-CAM dataset using inverse distance weighting (IDW), Kriging, CRAI and diffusion models. The two metrics were calculated for each time step individually and then averaged over the entire time span. The intensity of the cell color reflects the accuracy of the corresponding metric (the darker the better). Results shown in bold yellow correspond to the best values for each index and metrics.

	**TX90p**		**TX10p**		**TN90p**		**TN10p**	
Dataset	WD	R^2	WD	R^2	WD	R^2	WD	R^2
**IDW**	1.73	0.62	2.22	0.67	1.58	0.62	2.32	0.64
**Kriging**	1.77	0.66	2.24	0.70	1.64	0.64	2.30	0.67
**CRAI**	**0.99**	**0.75**	1.39	**0.77**	**1.02**	**0.71**	**1.56**	**0.73**
**Diffusion**	1.41	0.50	**1.35**	0.74	1.16	0.68	1.65	0.71

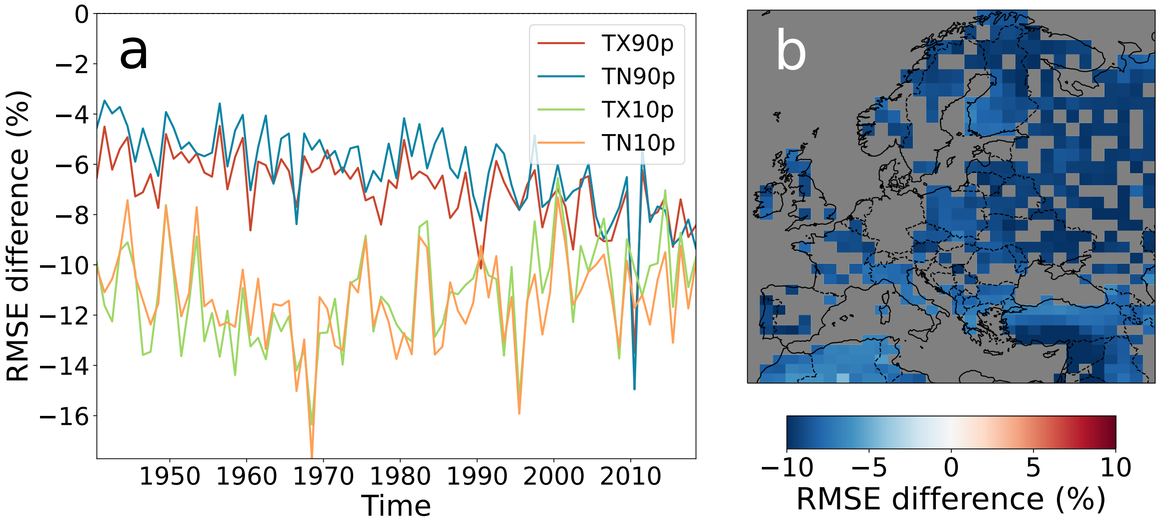

Figure S5: **Comparison of the temporal and spatial root mean square error (RMSE) between the CRAI reconstructions of ERA5 and HadEX3.** The two plots present the difference of the AI RMSE and HadEX3 RMSE with respect to the ERA5 dataset. a) shows the spatial mean of the RMSE difference for TX90p, TN10p, TX10p and TN10p, considering the reconstructed values only. TX90p is the percentage of days when the daily maximum temperature $> 90^{\text{th}}$ percentile. TX10p is the percentage of days when the daily maximum temperature $< 10^{\text{th}}$ percentile. TN90p is the percentage of days when the daily minimum temperature $> 90^{\text{th}}$ percentile. TN10p is the percentage of days when the daily minimum temperature $< 10^{\text{th}}$ percentile. b) shows the temporal mean of the RMSE differences averaged over the four extreme indices, considering the grid boxes with at least 10% of reconstructed values (otherwise, grid boxes are shown in gray). Positive values indicate a larger error in the AI reconstruction while negative values indicate a larger error for HadEX3.

Table S5: **Evaluation of the reconstruction methods using the ERA5 dataset.** The table shows the Wasserstein distance WD (left inner column) and the coefficient of determination R^2 (right inner column) calculated on the reconstructed values only for each extreme index and for four datasets: the reconstruction of a masked version of the HadEX-CAM dataset using inverse distance weighting (IDW), Kriging, CRAI and diffusion models. The two metrics were calculated for each time step individually and then averaged over the entire time span. The intensity of the cell color reflects the accuracy of the corresponding metric (the darker the better). Results shown in bold yellow correspond to the best values for each index and metrics.

	**TX90p**		**TX10p**		**TN90p**		**TN10p**	
Dataset	WD	R^2	WD	R^2	WD	R^2	WD	R^2
**HadEX3**	2.67	0.69	3.26	0.54	2.72	0.70	3.75	0.50
**IDW**	1.70	0.65	1.82	0.69	1.68	0.67	1.93	0.66
**Kriging**	1.60	0.69	1.67	0.72	1.64	0.70	1.77	0.70
**CRAI**	**1.04**	**0.76**	**1.08**	**0.78**	**1.07**	**0.76**	**1.23**	**0.75**
**Diffusion**	1.19	0.70	1.14	0.75	1.18	**0.76**	1.45	0.72

Table S6: **Evaluation of the reconstruction methods using the HadEX-CAM dataset.** The table shows the root mean square error RMSE (in %), the Spearman rank correlation coefficient SROCC, the Wasserstein distance WD and the coefficient of determination R^2 calculated on the reconstructed values only for each extreme index and for four datasets: the reconstruction of a masked version of the HadEX-CAM dataset using inverse distance weighting (IDW), Kriging, CRAI and diffusion models. The RMSE is computed for each dataset and index across all spatial and temporal data combined, whereas the SROCC, the WD, and the R^2 score are calculated for each time step individually and then averaged over the entire time span. Artificial missing values have been created in the HadEX-CAM dataset by applying the mask of missing values from January 1901 to all timesteps in the dataset. The intensity of the cell color reflects the accuracy of the corresponding metric (the darker the better). Results shown in bold yellow correspond to the best values for each index and metrics.

	**TX90p**		**TX10p**		**TN90p**		**TN10p**	
Dataset	RMSE	SROCC	RMSE	SROCC	RMSE	SROCC	RMSE	SROCC
**IDW**	6.86	0.62	7.37	0.64	5.86	0.62	7.12	0.65
**Kriging**	6.85	0.63	7.34	0.65	5.72	0.64	**6.91**	**0.67**
**CRAI**	6.71	**0.65**	**6.99**	**0.66**	5.67	**0.65**	**6.91**	0.66
**Diffusion**	**6.54**	0.64	7.16	0.65	**5.57**	**0.65**	7.18	0.65
	WD	R^2	WD	R^2	WD	R^2	WD	R^2
**IDW**	2.40	0.30	2.94	**0.29**	1.77	0.27	**2.59**	0.37
**Kriging**	3.23	0.31	3.67	**0.29**	2.42	0.31	3.12	**0.42**
**CRAI**	**2.03**	**0.33**	**2.48**	**0.29**	**1.59**	**0.34**	**2.59**	0.37
**Diffusion**	2.17	0.29	2.66	0.22	1.72	0.31	2.61	0.32

Table S7: **Evaluation of the reconstruction methods using a masked version of the ERA5 dataset.** The table shows the root mean square error RMSE (in %), the Spearman rank correlation coefficient SROCC, the Wasserstein distance WD and the coefficient of determination R^2 calculated on the reconstructed values only for each extreme index and for four datasets: the reconstruction of a masked version of the HadEX-CAM dataset using inverse distance weighting (IDW), Kriging, CRAI and diffusion models. The RMSE is computed for each dataset and index across all spatial and temporal data combined, whereas the SROCC, the WD, and the R^2 score are calculated for each time step individually and then averaged over the entire time span. Artificial missing values have been created in the HadEX-CAM dataset by applying the mask of missing values from January 1901 to all timesteps in the dataset. The intensity of the cell color reflects the accuracy of the corresponding metric (the darker the better). Results shown in bold yellow correspond to the best values for each index and metrics.

	**TX90p**		**TX10p**		**TN90p**		**TN10p**	
Dataset	RMSE	SROCC	RMSE	SROCC	RMSE	SROCC	RMSE	SROCC
**IDW**	5.90	0.76	6.38	0.74	5.01	0.81	5.57	0.80
**Kriging**	5.97	0.76	6.34	0.76	4.84	0.82	5.40	0.81
**CRAI**	5.55	**0.78**	**5.74**	**0.78**	4.45	0.84	**4.98**	**0.83**
**Diffusion**	**5.33**	**0.78**	5.78	0.77	**4.26**	**0.85**	5.21	0.82
	WD	R^2	WD	R^2	WD	R^2	WD	R^2
**IDW**	1.95	0.54	2.18	0.52	1.39	0.64	1.60	0.63
**Kriging**	2.61	0.53	2.76	0.53	1.78	0.65	1.94	0.65
**CRAI**	**1.57**	**0.56**	**1.67**	**0.57**	**1.13**	0.70	**1.33**	**0.68**
**Diffusion**	1.72	0.52	1.79	0.47	1.22	**0.72**	1.57	0.59

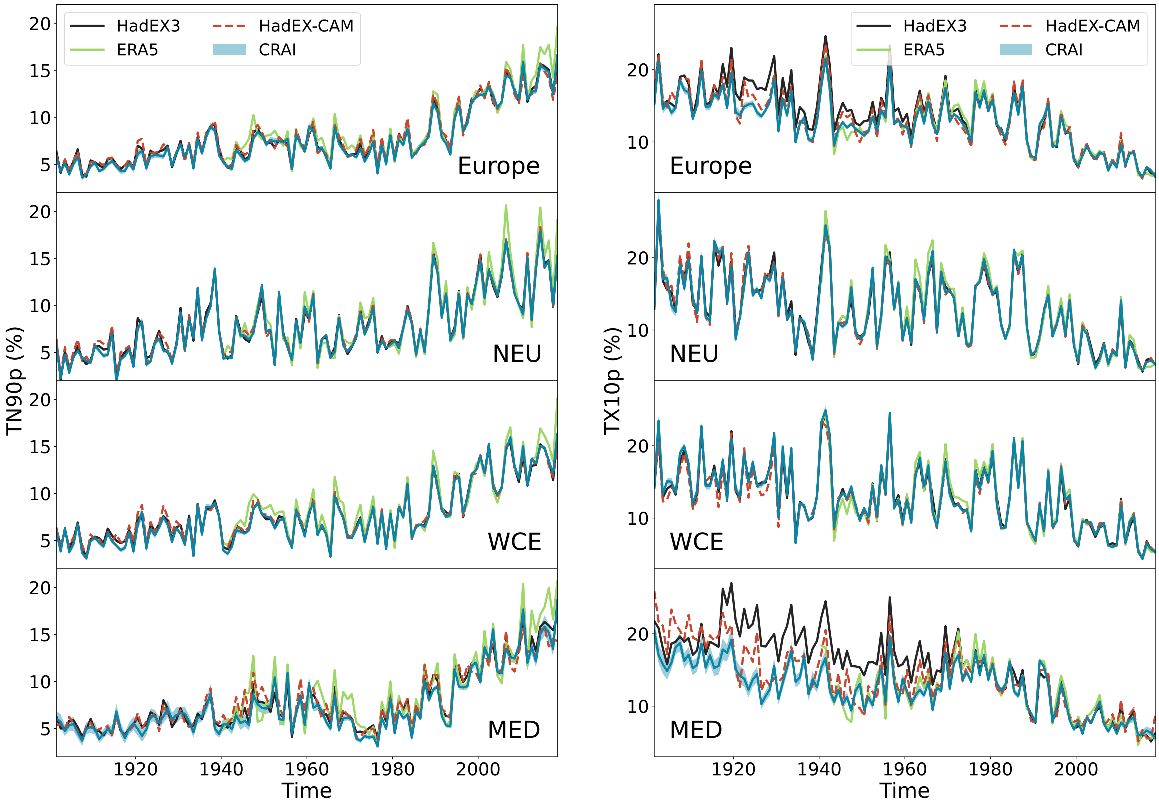

Figure S6: **Comparison of the regional means of TN90p and TX10p.** TN90p is the percentage of days when the daily minimum temperature > 90th percentile. TX10p is the percentage of days when the daily maximum temperature < 10th percentile. The original HadEX3 dataset is shown in black, the ERA5 dataset in green, the HadEX-CAM dataset in dashed red, and its reconstruction using CRAI in blue. The min/max spread of the twenty reconstructions is shown as a semi-transparent blue area and the mean of the reconstructions is shown as a solid blue line. Spatial means are calculated for the full grid (top panel) and for three European regions defined by the AR6 IPCC report ([1]): Northern Europe (NEU), Western and Central Europe (WCE), Mediterranean(MED).

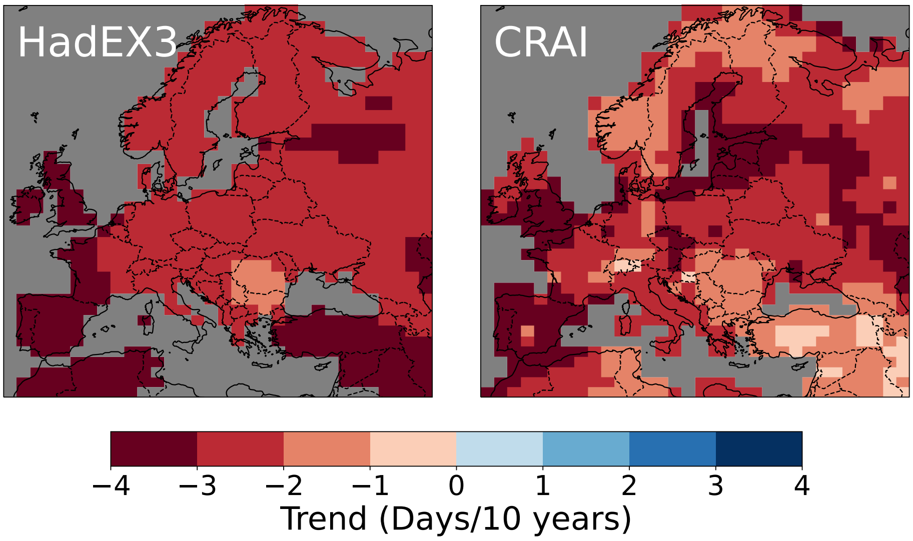

Figure S7: **Linear trends (in days/10 years) of TX10p for the period 1901-2018.** TX10p is the percentage of days when the daily maximum temperature < 10th percentile. Left panel: original HadEX3 dataset (considering only grid boxes with at least 66% of valid data across the whole time period). Right panel: AI reconstruction.

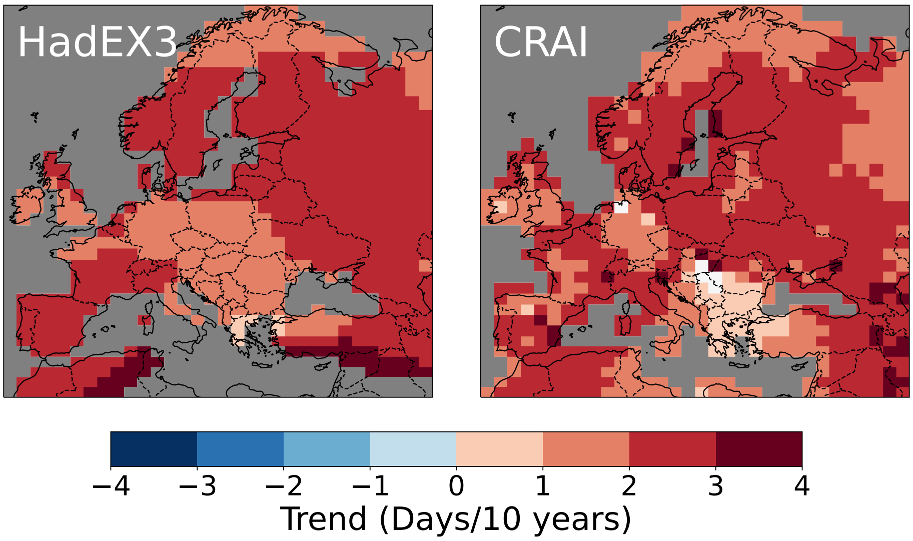

Figure S8: **Linear trends (in days/10 years) of TN90p for the period 1901-2018.** TN90p is the percentage of days when the daily minimum temperature > 90th percentile. Left panel: original HadEX3 dataset (considering only grid boxes with at least 66% of valid data across the whole time period). Right panel: AI reconstruction.

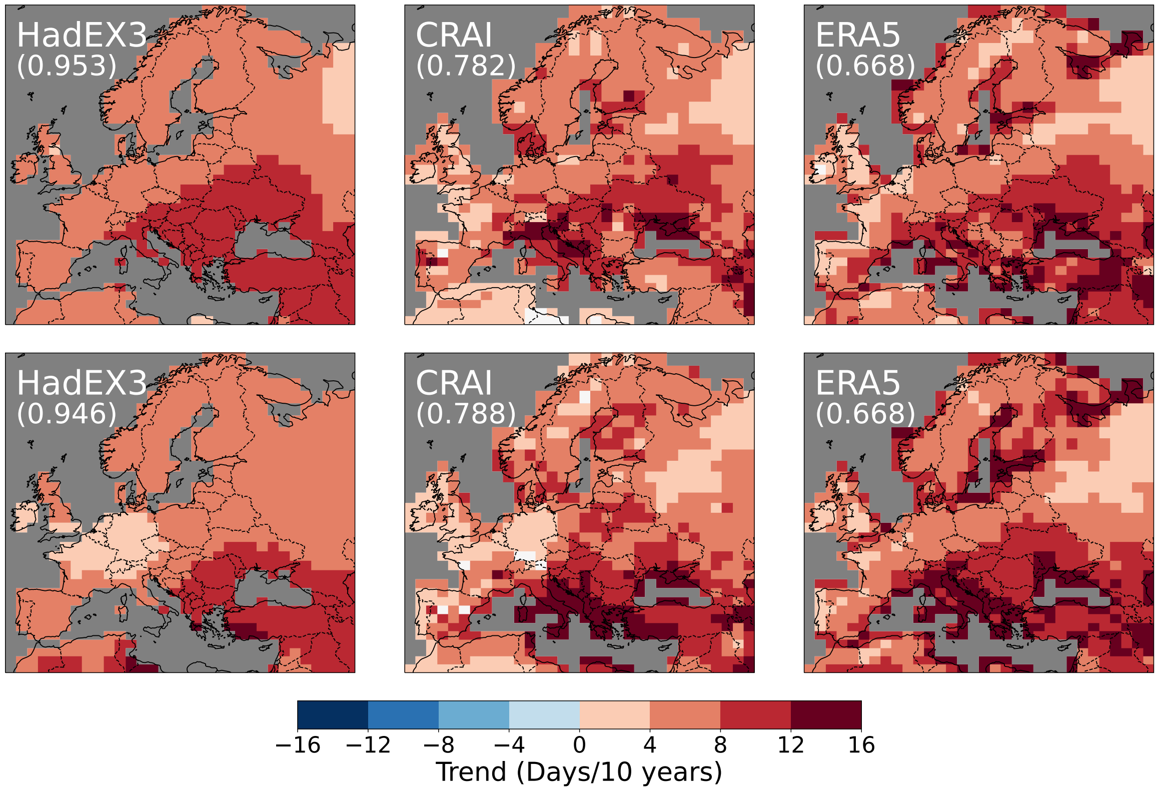

Figure S9: **Linear trends (in days/10 years) of TX90p (first row) and TN90p (second row) for the period 1980-2018.** TX90p is the percentage of days when the daily maximum temperature $> 90^{\text{th}}$ percentile. TN90p is the percentage of days when the daily minimum temperature $> 90^{\text{th}}$ percentile. Left panels: original HadEX3 dataset (considering only grid boxes with at least 66% of valid data across the whole time period). Central panels: AI reconstruction. Right panels: ERA5. The number in parenthesis indicates the global Moran's I value for each dataset considering only the direct neighbors of each gridbox.

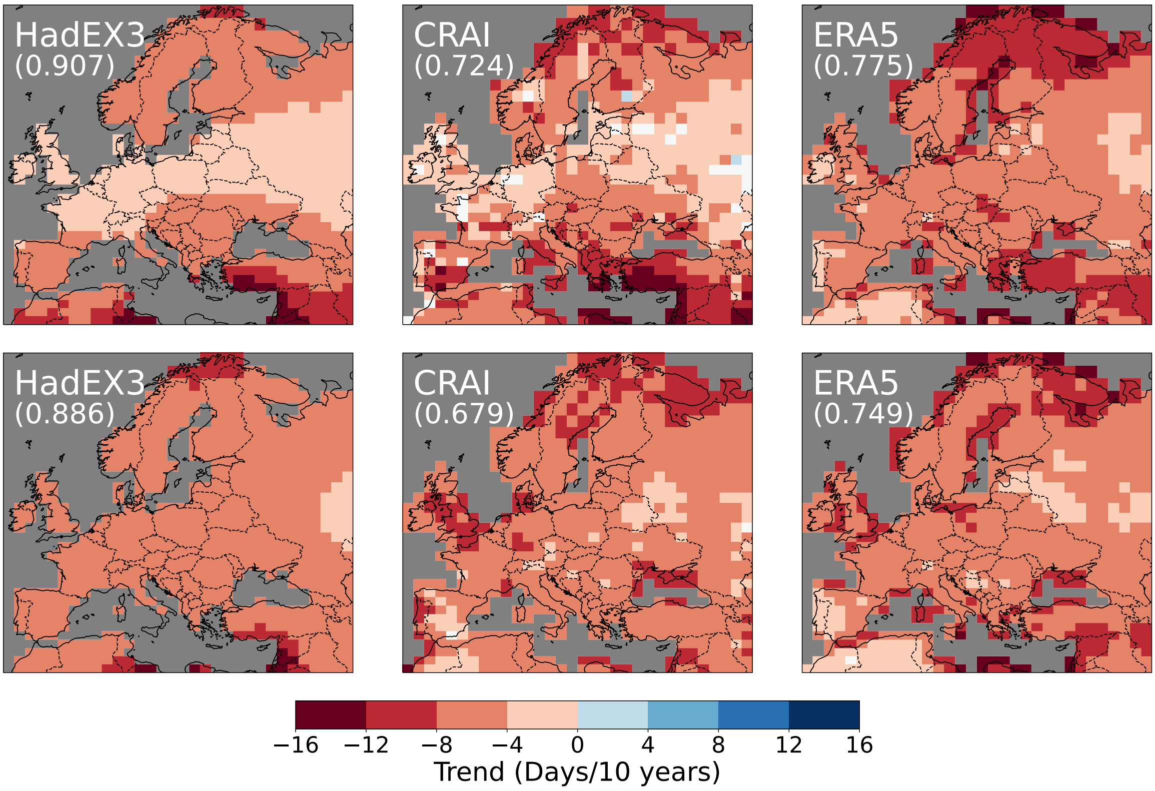

Figure S10: **Linear trends (in days/10 years) of TN10p (first row) and TX10p (second row) for the period 1980-2018.** TN10p is the percentage of days when the daily minimum temperature $< 10^{\text{th}}$ percentile. TX10p is the percentage of days when the daily maximum temperature $< 10^{\text{th}}$ percentile. Left panels: original HadEX3 dataset (considering only grid boxes with at least 66% of valid data across the whole time period). Central panels: AI reconstruction. Right panels: ERA5. The number in parenthesis indicates the global Moran's I value for each dataset considering only the direct neighbors of each gridbox.

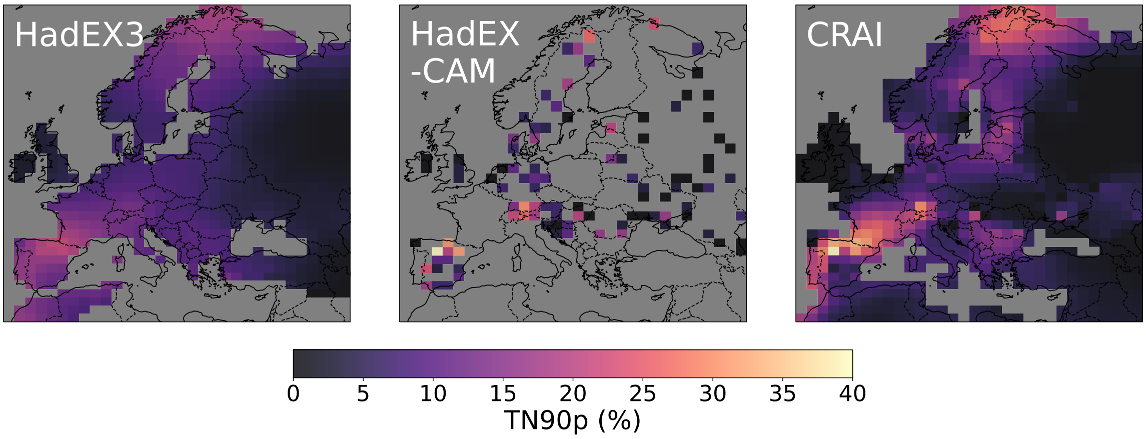

Figure S11: **TN90p for a reported heatwave event (September 1911).** TN90p is the percentage of days when the daily minimum temperature $> 90^{\text{th}}$ percentile. Left panel: original HadEX3 dataset. Central panel: original HadEX-CAM dataset. Right panel: AI reconstruction.

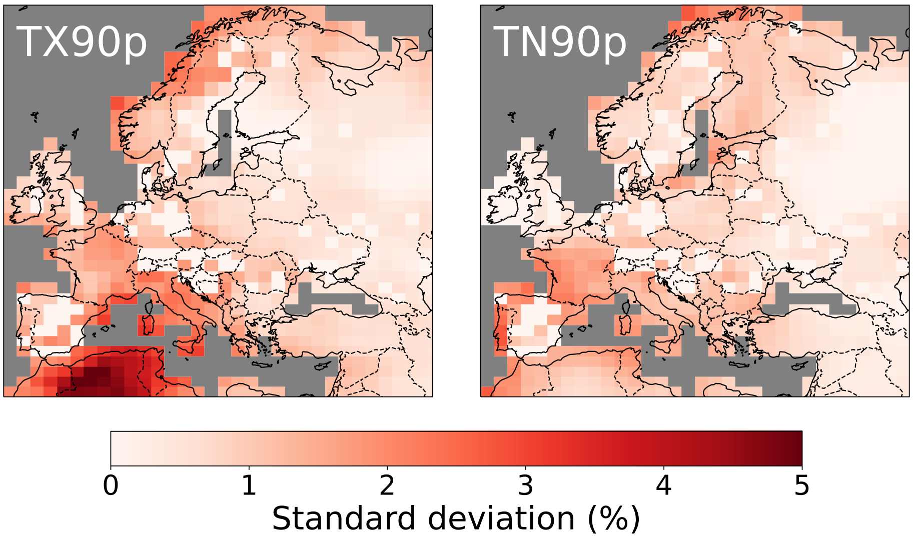

Figure S12: **Standard deviation (in %) of the CRAI reconstructions of TX90p and TN90p from HadEX-CAM for a reported heatwave event (September 1911).** TX90p is the percentage of days when the daily maximum temperature $> 90^{\text{th}}$ percentile. TN90p is the percentage of days when the daily minimum temperature $> 90^{\text{th}}$ percentile.

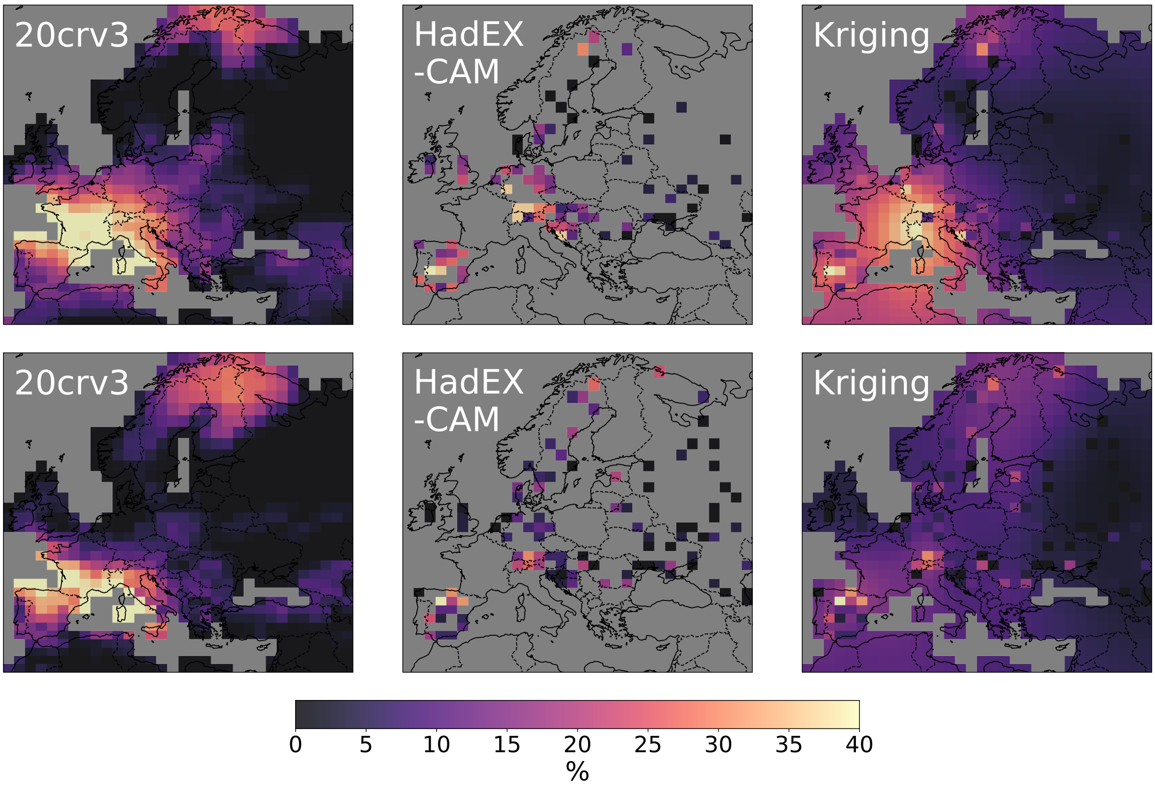

Figure S13: **TX90p (first row) and TN90p (second row) for a reported heatwave event (September 1911).** TX90p is the percentage of days when the daily maximum temperature $> 90^{th}$ percentile. TN90p is the percentage of days when the daily minimum temperature $> 90^{th}$ percentile. Left panels: 20th Century Reanalysis Version 3. Central panels: Original HadEX-CAM. Right panels: Kriging reconstruction.

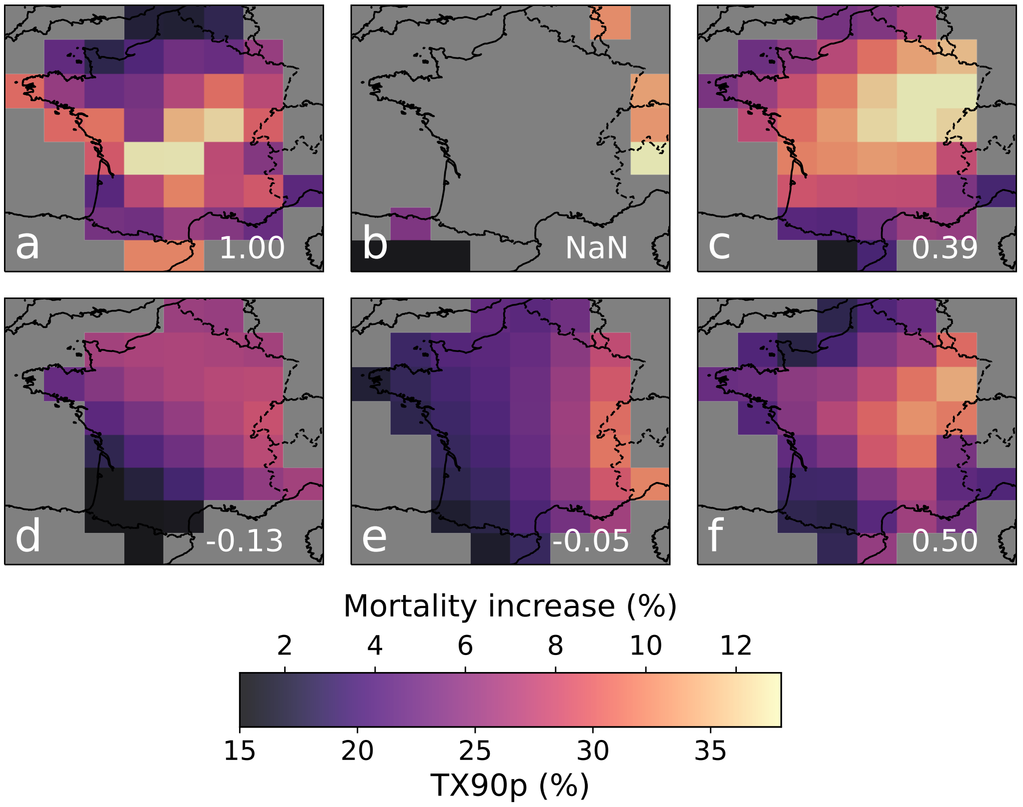

Figure S14: **Comparison between the mortality of seniors in France in 1911 and the TX90p values for several datasets.** TX90p is the percentage of days when the daily maximum temperature > 90th percentile. a) Increase in percentage of the mortality of persons older than 65 years in 1911 with respect to the mean value of 1909, 1910, 1912, 1913. The data have been created following the methodology described in [2, 3] and have been provided by the authors. The data built at the departmental level have been regridded to the HadEXCAM grid by area-weighted interpolation. The remaining panels correspond to the TX90p values obtained by averaging across the months spanning the duration of the heatwave (July, August and September): b) Original HadEX-CAM dataset, c) AI reconstruction of HadEX-CAM, d) HadEX3, e) Kriging reconstruction of HadEX-CAM, e) 20cr-v3 dataset. The number on the bottom right corner of each panel indicates the Spearman rank correlation coefficient (SROCC) of each dataset with the normalized data in a).

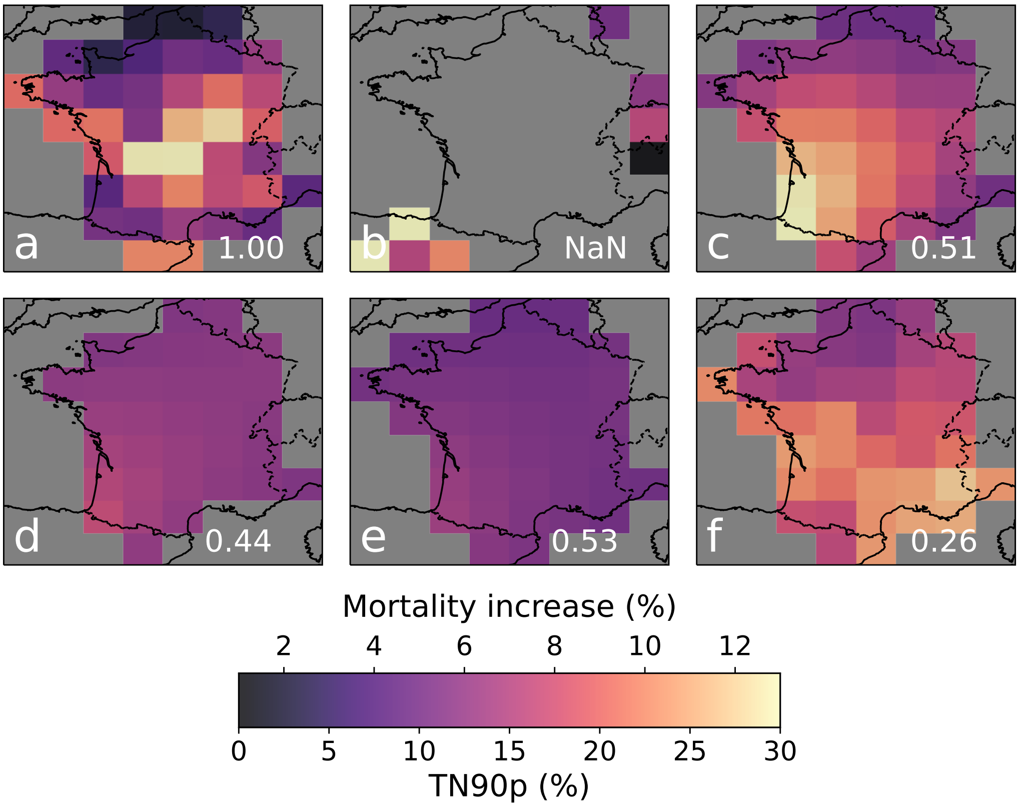

Figure S15: **Comparison between the mortality of seniors in France in 1911 and the TN90p values for several datasets.** TN90p is the percentage of days when the daily minimum temperature > 90th percentile. a) Increase in percentage of the mortality of persons older than 65 years in 1911 with respect to the mean value of 1909, 1910, 1912, 1913. The data have been created following the methodology described in [2, 3] and have been provided by the authors. The data built at the departmental level have been regridded to the HadEXCAM grid by area-weighted interpolation. The remaining panels correspond to the TX90p values obtained by averaging across the months spanning the duration of the heatwave (July, August and September): b) Original HadEX-CAM dataset, c) AI reconstruction of HadEX-CAM, d) HadEX3, e) Kriging reconstruction of HadEX-CAM, e) 20cr-v3 dataset. The number on the bottom right corner of each panel indicates the Spearman rank correlation coefficient (SROCC) of each dataset with the normalized data in a).

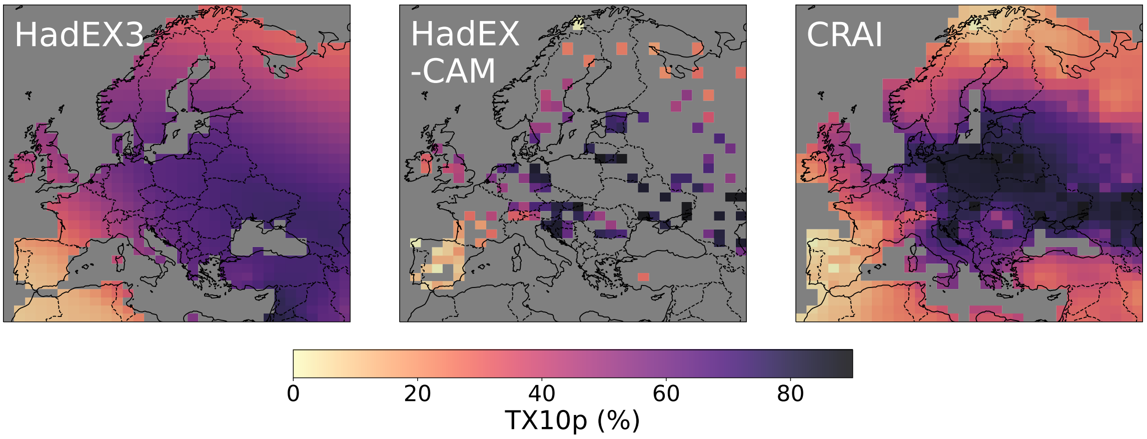

Figure S16: **TX10p for a reported coldwave event (February 1929).** TX10p is the percentage of days when the daily maximum temperature < 10th percentile. Left panel: original HadEX3 dataset. Central panel: original HadEX-CAM dataset. Right panel: AI reconstruction.

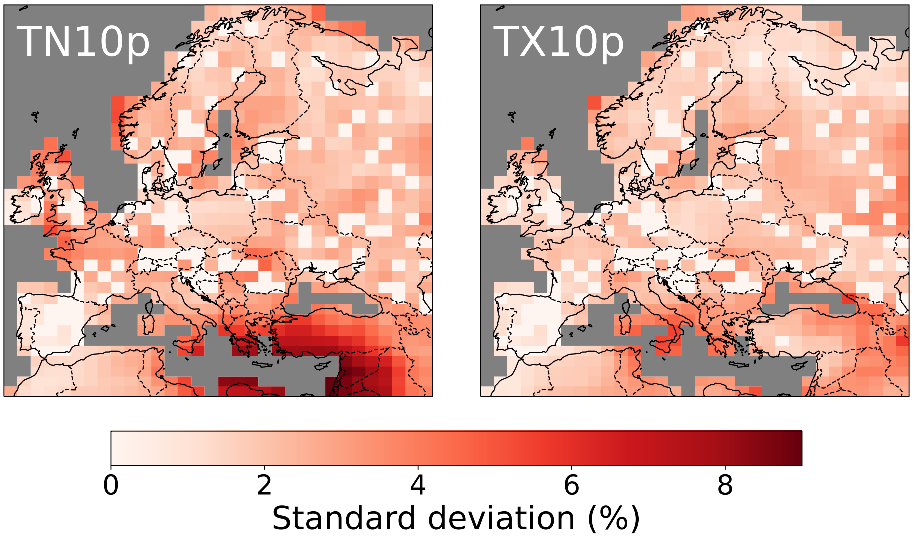

Figure S17: **Standard deviation (in %) of the CRAI reconstructions of TN10p and TX10p from HadEX-CAM for a reported coldwave event (February 1929).** TN10p is the percentage of days when the daily minimum temperature < 10th percentile. TX10p is the percentage of days when the daily maximum temperature < 10th percentile.

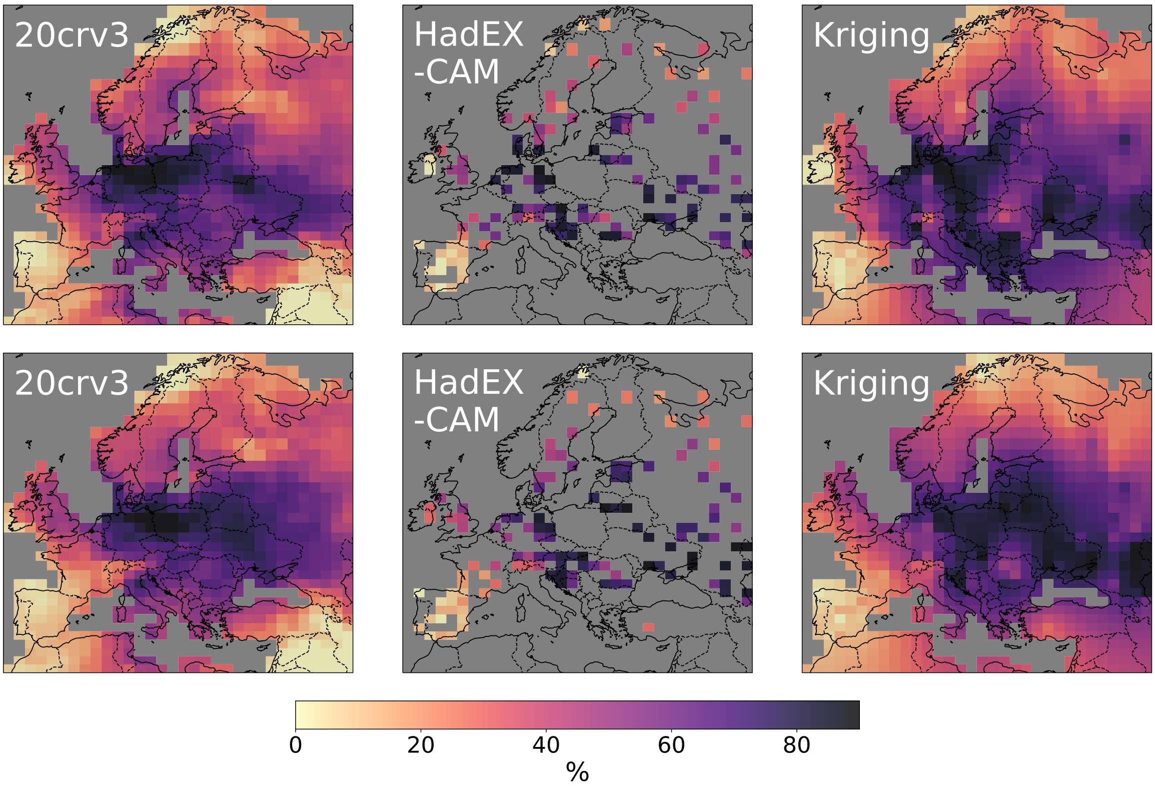

Figure S18: **TN10p (first row) and TX10p (second row) for a reported coldwave event (February 1929).** TN10p is the percentage of days when the daily minimum temperature $< 10^{\text{th}}$ percentile. TX10p is the percentage of days when the daily maximum temperature $< 10^{\text{th}}$ percentile. Left panels: 20th Century Reanalysis Version 3. Central panels: Original HadEX-CAM. Right panels: Kriging reconstruction.

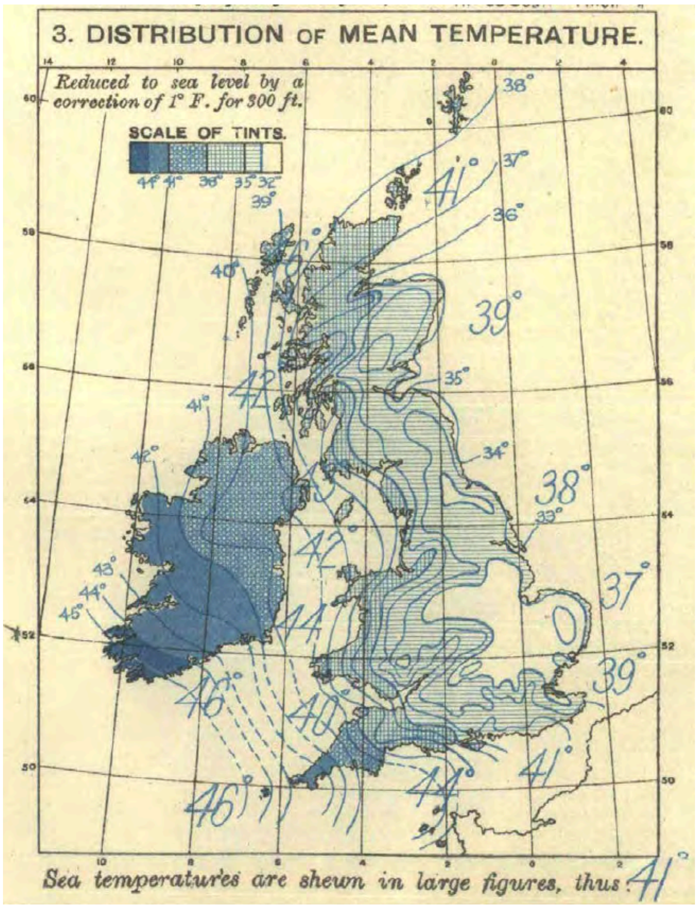

Figure S19: **Mean temperatures (in Fahrenheit) from the UK monthly weather report (Met Office), February 1929, panel 3 on page 17,** `https://digital.nmla.metoffice.gov.uk/IO_4836bad7-1538-4ab0-8bd1-7cee6bfc3c55`. Information provided by the National Meteorological Library and Archive – Met Office, UK.

Supplementary References

[1] Maialen Iturbide et al. "An update of IPCC climate reference regions for subcontinental analysis of climate model data: definition and aggregated datasets". In: *Earth Syst. Sci. Data* 12.4 (2020), pp. 2959–2970. ISSN: 18663516. DOI: 10.5194/essd-12-2959-2020.

[2] Florian Bonnet. "Computations of French lifetables by department, 1901–2014". In: *Demogr. Res.* 42.26 (2020), pp. 741–762. DOI: 10.4054/DemRes.2020.42.26.

[3] Florian Bonnet and Hippolyte d'Albis. "Spatial Inequality in Mortality in France over the Past Two Centuries". In: *Popul. Dev. Rev.* 46.1 (2020), pp. 145–168. DOI: https://doi.org/10.1111/padr.12318.
